# Designing a unique feedback mechanism for hydrogel-forming microneedle array patches: a concept study

**DOI:** 10.1007/s13346-021-01033-1

**Published:** 2021-07-31

**Authors:** Aaron R. J. Hutton, Melissa Kirkby, Eneko Larrañeta, Ryan F. Donnelly

**Affiliations:** School of Pharmacy, Queen’s University Belfast, 97 Lisburn Road, Belfast, BT9 7BL UK

**Keywords:** Microneedle array patch (MAP), Water-filled polymeric reservoir (PR), Feedback mechanism, Skin insertion

## Abstract

Although microneedle array patch (MAP) technology is reaching ever closer to regulatory approval, it remains imperative that approaches to further improve patient acceptance are still explored. Addressing this perception, a water-filled reservoir was incorporated into a hydrogel-forming MAP system to provide a novel feedback mechanism. To confirm successful MAP skin insertion, the end user would both hear and feel the rupture of the water-filled reservoir. Interestingly, a 50-µL water-filled reservoir ruptured at 30.27 ± 0.39 N, which has previously been shown as the mean application force for MN insertion in human subjects following appropriate instruction. Importantly, no significant difference in % cumulative permeation of FITC-dextran 10 kDa and fluorescein sodium after 24 h was observed between a 50-µL reservoir and the current method of application that has been successfully used in both in vitro and in vivo studies (*p* > 0.05). Therefore, as drug delivery was not affected, this proof-of-concept study has shown that a water-filled reservoir feedback mechanism has the potential to serve as a viable tool for consistent MAP skin insertion.

**Graphical abstract:**

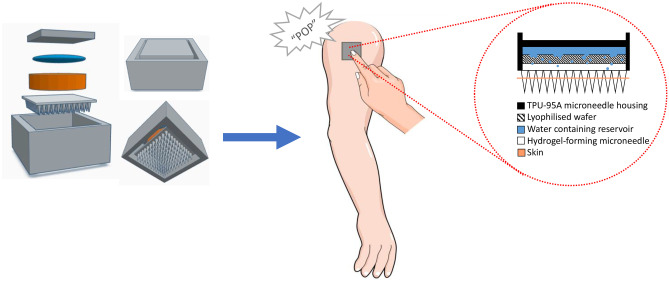

## Introduction

Microneedle array patch (MAP) delivery systems have been exploited for many years, achieving considerable success within the cosmetic industry [1–4]. Unfortunately, no MAP has gained FDA approval for the delivery of therapeutic compounds, however, recent collaborations with academic institutions and pharmaceutical manufacturers is thought to change this trend [5–7]. In particular, Zosano Pharma is currently seeking approval for an MAP indicated for migraine treatment [8]. If approved, it is widely regarded that this exemplar product will encourage other pharmaceutical companies to exploit this emerging market. In addition, the COVID-19 pandemic has emphasised that there is clear need for self-administered systems that do not require the presence of a healthcare professional. Indeed, MAP systems have the potential to accelerate vaccination campaigns by enabling patients to administer their vaccine in the comfort and safety of their own homes, reducing the burden on healthcare facilities.

Despite the absence of therapeutic MAPs on the market, qualitative studies have shown that the views and opinions of MAPs remains positive [9]. Both healthcare professionals and the public have expressed a preference for pain-free, self-administered MAPs when compared to a traditional hypodermic syringe and needle [10, 11]. Appreciating these benefits, the Program for Appropriate Technology in Health (PATH), a non-profit global health organisation, judges MAPs to be of particular benefit in low- and middle-income countries [12]. This includes the delivery of both vaccines and essential medicines. Dissolving and hydrogel-forming MAPs, which are self-disabling by design, have real potential within this area, as they eliminate the need for cold chain storage, do not require a healthcare professional for administration and do not require sharps disposal [13–15].

Previous studies have shown that MAPs can be self-applied with minimal assistance, only requiring thumb pressure to achieve complete skin insertion [16]. Nevertheless, participants within these studies expressed a preference for a feedback mechanism to confirm that the correct force had been applied for successful skin insertion. Using a stainless-steel MAP for influenza vaccination, the first feedback mechanism developed used a snap-based device that closed shut at a certain force [17]. The usability of this device was comparable with the usability rates of the oral typhoid vaccine, the only self-administered vaccine approved within the USA [18]. This suggests that a MAP coupled with an insertion force feedback mechanism has the potential to provide a reliable self-vaccination method. Vicente-Pérez et al. have also evaluated an insertion feedback mechanism for the more recently developed hydrogel-forming MAPs. In this study, a low-cost pressure indicating sensor film was used to successfully provide visual feedback to the user to confirm correct application [19]. Therefore, by developing strategies to further enhance the usability of a MAP, both studies have certainly enabled this technology to move step closer market.

In this proof-of-concept study, a water-filled reservoir has been incorporated into a hydrogel-forming MAP system to provide a skin insertion feedback mechanism. This particular reservoir was designed to fracture at the required force for successful MAP application, with the point of rupture both felt and heard by the patient. This unique feedback design was explored further by examining the effect of a burst release of water on in vitro permeation using two model compounds, fluorescein sodium and FITC-dextran 10 kDa, using ‘super-swelling’ MAPs. The composition of these particular MAPs is based upon ‘swellable’ MAPs, the first hydrogel formulation documented in the literature [20]. The addition of Na_2_CO_3_ results in the formation of sodium salts on free carboxylic acid groups, generating a more porous structure and increasing the degree of swelling. Therefore, the lower density and greater space within the polymer itself permits greater permeation of higher MW macromolecules such as FITC-dextran 10 kDa examined in this study [21]. By designing an alternative method to improve the applicability of hydrogel-forming MAPs, this unique study could have important implications for future patient acceptance.

## Materials and methods

### Materials

Elastollan® thermoplastic polyurethane pellets with 95A shore hardness (TPU 95A) was generously supplied by DistruPol Ltd, Dublin, Ireland. Fluorescein isothiocyanate–dextran (FITC-dextran 10, MW = 8000–12,000 Da) was purchased from TdB Consultancy AB (Uppsala, Sweden). Fluorescein sodium (MW = 376 Da) and poly(ethylene glycol) (PEG) with MW = 10 000 Da were purchased from Sigma-Aldrich, Dorset, UK. Gantrez® S-97, a co-polymer of methyl vinyl ether and maleic acid (PMVE/MA) was a gift from Ashland, Kidderminster, UK. Sodium carbonate (Na_2_CO_3_) was purchased from BDH Laboratory Supplies, London, UK. Pearlitol® 50C-Mannitol was supplied by Roquette, Lestrem, France. Cryogel SG3 was provided by PB Gelatins, Pontypridd, UK.

### MAP housing concepts and fabrication using additive manufacturing

MAP housing concepts were designed and developed using computer-aided design (CAD)–based software. A number of different factors were considered during the design process, including ease of use, water retention ability, ease of manufacture, reservoir piercing ability and protective capabilities during transportation. Once the final design was chosen, the MAP hollow housing and lid were printed with an Ultimaker3 3D printer (Ultimaker, Geldermalsen, The Netherlands) with Cura® software. Using one 0.4-mm nozzle on the Ultimaker3 system, TPU 95A was extruded with a print speed of 25 mm/s and a print temperature of 215 °C. The infill layer thickness was 0.1 mm.

### Fabrication of Parafilm^®^ M water-containing reservoirs

**Fig. 1:**
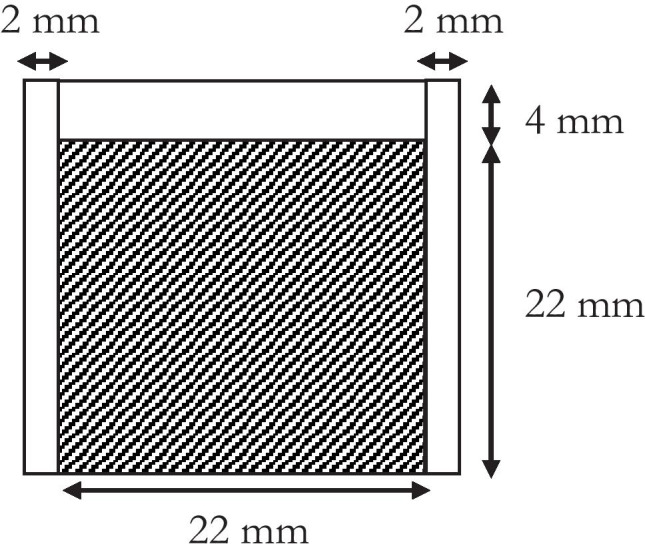
Schematic diagram of Parafilm® M water-filled reservoirs, with the shaded region representing the water-filled compartment

Parafilm® M reservoirs (PR) were produced using a heat-sealing method. In particular, individual sheets of Parafilm® M were cut to size (60 × 26 mm) and folded over once. The two adjacent sides, with a width of 2 mm, were then heat sealed for 10 s using a 400-W Packer P400/C impulse heat sealer (Packaging Aids Ltd, King’s Lynn, Norfolk) with a maximum sealing temperature of 215 °C. A defined volume of water (50 μL, 200 μL or 600 μL) was dispensed into PR, named PR 50, PR 200 and PR 600, respectively. The top opening was then folded over and heat-sealed, thus producing a water-filled polymeric reservoir (Fig. 1).

### Fracture force and water release from Parafilm® M reservoirs

The volume of water released from PR 50, PR 200 and PR 600 was investigated using a TA.XT Texture Analyser (Stable Micro Systems, Surrey, UK) in compression mode. Each individual reservoir was folded once, enclosed inside the MAP housing and lid and then placed on the Texture Analyser platform. A moveable cylindrical probe (cross-sectional area 1.5 cm^2^) moved vertically downward at a rate of 1.19 mm/s onto the 3D printed lid. In doing so, the reservoirs ruptured, enabling the fracture force to be determined. To assess the volume of water released, the reservoir and lid were then removed from the weigh boat and the mass recorded (Eq. 1).

1 $$Volume\;of\;water\;released\;\left(\mu L\right)=\frac{M_a-M_b}\rho$$

*M*_a_ = Mass after compression.

*M*_b_ = Mass before compression.

*ρ* = Density (0.998 g/mL) at 20 °C.

### Manufacture of FITC-dextran and fluorescein sodium lyophilised wafers

FITC-dextran (10 kDa) and fluorescein sodium–loaded lyophilised wafers were prepared using 10% w/w gelatin and 5% w/w mannitol as detailed by Hutton et al. [21]. Briefly, the fluorescent compound was first dissolved in deionised water and heated to 37 °C. Mannitol and gelatin were then added, followed by hand mixing to facilitate dissolution. The formulations were then sonicated at 37 °C for 60 min before casting into open ended moulds with diameter 11.8 mm and depth 2 mm. Each formulation was frozen at −80 °C for 60 min and then placed in a Virtis™ Advantage XL-70 bench top lyophiliser (SP Scientific®, PA, USA). Using an adapted version of a previously described method, the freeze drying process involved: primary drying for 90 min at a shelf temperature of −40 °C, 90 min at a shelf temperature of −30 °C, 90 min at a shelf temperature of − 20 °C, 530 min at a shelf temperature of −10 °C and 90 min at a shelf temperature of 0–10 °C (Donnelly et al. [22]). The secondary drying phase was performed over 660 min at a shelf temperature of 25 °C and a vacuum pressure of 50 mTorr.

### Fabrication of ‘super-swelling’ hydrogel–forming MAPs

‘Super-swelling’ hydrogel–forming MAPs were formulated from an aqueous blend of 20% w/w Gantrez® S-97, 7.5% w/w PEG 10,000 and 3% w/w Na_2_CO_3_ as described previously [23]. Briefly, an aqueous blend was obtained by firstly, fully dissolving Gantrez® S-97 and PEG 10,000 in water; then, Na_2_CO_3_ was added. The blend was mixed well until foaming had ceased and a uniform gel was formed. The formulation was then centrifuged for 15 min at 3500 rpm to remove air bubbles. The formulation was dispensed onto prefabricated silicone moulds: each mould had an array of 11 × 11 conical needles, each either 400 μm or 600 μm high and 300 μm at the base with an interspacing of 50 μm. The hydrogel-forming MAPs were allowed to dry within the silicon moulds at room temperature for 24 h, after which the MAPs were removed from their moulds, sidewalls were removed using a sharp scalpel blade and the arrays were cross-linked through an esterification reaction by heating at 80 °C for a further 24 h.

### ‘Super-swelling’ hydrogel–forming MAP insertion into a polymeric film using the unique feedback design

The insertion capabilities of two ‘super-swelling’ hydrogel-forming MAPs, composed of 400-μm and 600-μm needle heights respectively and contained within the 3D printed housing, were assessed using eight layers of Parafilm® M. This polymeric material has previously shown its capabilities as a suitable model for human skin (Larrañeta et al. [24, 25]). For comparison, three separate setups were used, namely MAP alone, MAP with lyophilised wafer situated on top of the MAP baseplate and MAP inside the 3D printed housing to determine whether the addition of the water-filled reservoir would adversely affect MAP insertion. With the latter, a hydrogel-forming MAP was placed inside the 3D printed housing. A FITC-dextran/fluorescein sodium lyophilised wafer was then placed on top of the MAP, followed by a Parafilm® M water-filled reservoir. A 3D printed lid was subsequently set on top to secure the three separate components and to prevent the loss of water following the rupture of the polymeric reservoir. Using a TA.XT Texture Analyser in compression mode, a force of 30 N was applied to the 3D printed lid for 30 s using a moveable cylindrical probe as mentioned previously. To assess insertion, each Parafilm® M layer was examined using a Leica EZ4W stereo microscope and the number of holes counted. Insertion in each layer is regarded as successful if the number of holes created in each layer is greater than 20% [24].

### Ex vivo ‘super-swelling’ hydrogel–forming MAP insertion into full-thickness porcine skin using the all-in-one design

Full-thickness neonatal porcine was used to examine the insertion capabilities of a ‘super-swelling’ MAP within a biological matrix using the one-step MAP insertion method. In this instance, the 3D printed housing contained PR 50, PR 200 or PR 600 and a ‘super-swelling’ MAP. The FITC-dextran/fluorescein sodium containing wafer was removed to minimise interference during imaging. Using the applicator device, each MAP was inserted into the skin using manual thumb pressure for 30 s. Importantly, this force had the ability to fracture the water containing reservoir. Optical coherence tomography (OCT) imaging was used to assess the MAPs insertion and swelling capacity following the addition of water after 24 h, expressed in terms of MAP base plate diameter (µm) and base plate/*stratum corneum* distance (µm).

### In vitro MAP swelling capacity using water-containing reservoirs

The swelling capacity of ‘super-swelling’ MAPs in vitro using PR 50, PR 200 and PR 600 was assessed using modified Franz diffusion cells. Each receiver vessel was filled with phosphate buffered saline (PBS) (pH 7.4), agitated at 600 rpm using a metal stir bar and equilibrated to 37 ± 1 °C. Neonatal porcine skin samples were shaved and fixed to the donor compartment using cyanoacrylate glue. Using a one-step insertion procedure, each MAP and water containing reservoir were inserted into dermatomed (350 µm) neonatal porcine skin using manual thumb pressure for 30 s. A stainless-steel weight (5 g) was placed on top and left in place for 24 h. The mass of each MAP was recorded before and after insertion, with the swelling capacity represented by the % mass increase after the 24-h period.

### In vitro permeation using the MAP feedback system

FITC-dextran and fluorescein sodium permeation through dermatomed (350 µm) neonatal porcine skin was quantified using modified Franz diffusion cells as detailed above. In short, using a one-step insertion procedure, MAPs, 600 μm in height, were placed into the centre of each donor compartment. A lyophilised wafer containing the compound of interest was placed on top. Water-filled reservoirs (PR 50, PR 200, PR 600) were folded once and placed above the lyophilised wafer. A 5.0-g stainless steel weight was placed on top and manual pressure applied for 30 s. As a comparison, MAP insertion in vitro using the traditional approach was used. This included the placement of an MAP into the centre of each donor compartment followed by the application of manual pressure for 30 s. A defined volume of water (10 µL) was then dropped onto the MAP baseplate to permit adhesion of the drug containing wafer, after which a 5.0-g stainless steel weight was placed on top. In both cases, the donor compartment was carefully placed on top of the receiver compartment and securely clamped into place. The sampling arm was occluded with Parafilm® M to prevent evaporation. Samples (200 µL) were removed at specified time points and diluted appropriately in PBS (pH 7.4). An equal volume of fresh pre-warmed PBS (pH 7.4) was added to the receiver vessel to replace this. Samples were analysed using fluorescence spectroscopy.

Similarity factor, *f*_2_, a statistical test typically used to compare dissolution profiles of solid dosage forms, was used to compare the permeation profiles of each MAP setup tested in vitro [26, 27]*.* Using this model independent approach, two cumulative permeation curves can be considered comparable when the *f*_2_ value is larger than 50 [28, 29]. The similarity factor (Eq. 2) is a logarithmic transformation of the sum-squared error of differences between the % cumulative permeation of the test, *T*_t_ and reference *R*_t_ over all timepoints, *n*.

2 $${f}_{2}=50.\mathrm{log}\left\{{\left[1+ \left(\frac{1}{n}\right)\sum_{j=1}^{n}{\left({R}_{t}-{T}_{t}\right)}^{2}\right]}^{-0.5}.100\right\}$$

### Pharmaceutical analysis of FITC-dextran 10 kDa and fluorescein sodium

Fluorescence analysis was carried out using a BMG FLUOstar Omega Fluorescence Microplate Reader (BMG LabTech, Ortenberg, Germany) at an excitation wavelength 485 nm and emission wavelength 520 nm. Standards of FITC-dextran 10 kDa were prepared in PBS (pH 7.4) within the range of 0.78 to 100 µg/mL. Fluorescein sodium standards were prepared in the range of 0.08 to 2.5 µg/mL. Least squares linear regression analysis and correlation analysis over three consecutive days was used to calculate the coefficient of determination (*R*^2^) and line equation. Limit of detection (LoD) and limit of quantification (LoQ) were calculated using Eqs. 3 and 4, with *S* defined as the gradient of the calibration curve and σ the standard deviation of the response, determined using the standard error of the *y*-intercepts on the regression line.

3 $$LoD=\frac{3.3\;\sigma}S$$

4 $$LoQ=\frac{10\;\sigma}S$$

### Statistical analysis

Statistical analysis was performed using GraphPad Prism version 8 (GraphPad Software Inc., San Diego, CA). This included calculations of the means and standard deviations, with a one-way analysis of variance (ANOVA) used to assess whether there were significant differences between the means of the data sets, with *p* < 0.05 denoting statistical significance.

## Results

### MAP housing concepts using CAD

**Fig. 2:**
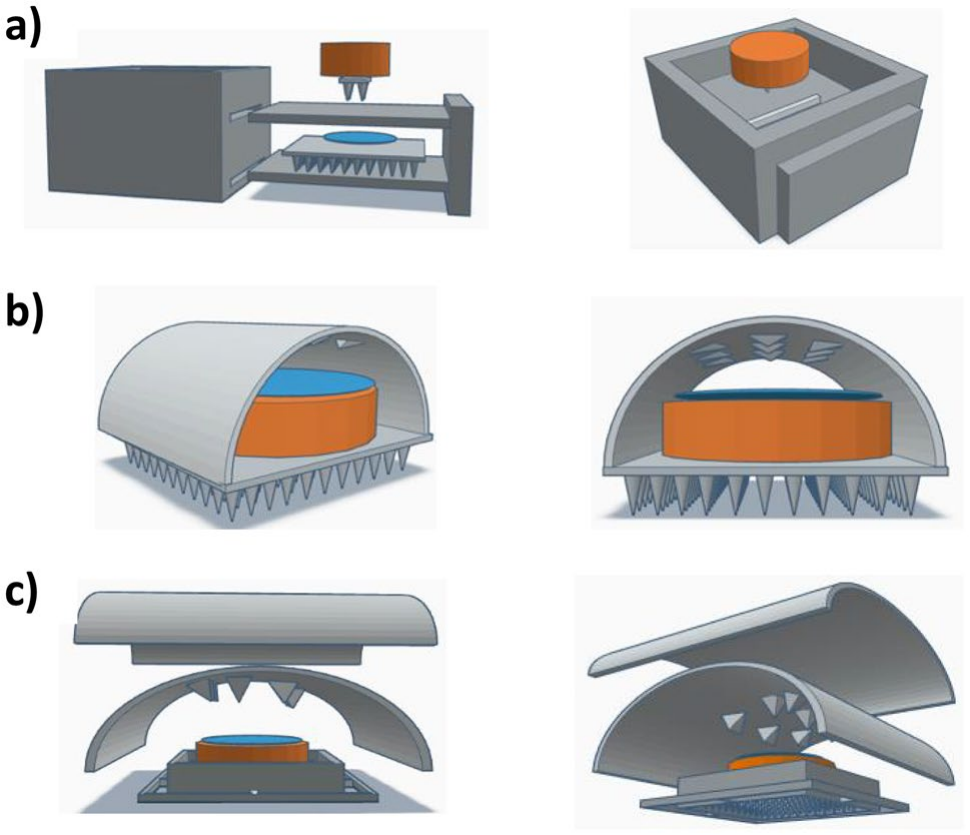
**a** Double shelf design (concept 1), **b** tunnel backing design (concept 2), **c** double backing design (concept 3)

Initially, three different MAP housing concepts were considered as shown in Fig. 2. The double shelf design (Fig. 2a) consisted of a hollow housing with 2 slits on one side, permitting the insertion of a double shelf secondary component. A MAP and water-filled reservoir would be placed on the lower shelf, with a dissolving MAP attached to a lyophilised wafer placed on the upper shelf. The concept in Fig. 2b consisted of a lyophilised wafer situated on top on the MAP, with a water-filled reservoir placed above the wafer. A 3D printed needle containing an Elastollan® sheet would be placed above the wafer and fixed into place on the MAP baseplate. To help retain water above the MAP, a second, flexible polymeric sheet placed in perpendicular orientation to the 3D printed flexible sheet as mentioned previously could be used as shown in (Fig. 2c). Rather than fixing the flexible backing sheets to the MAP baseplate, this design consisted of a MAP housing onto which both flexible sheets adhered to. However, manufacturing both concepts 2 and 3 could prove challenging, as adhering Elastollan® to either the MAP baseplate or MAP would be difficult to achieve. In addition, due to the two-step process associated with concept 1, end user acceptability could limit its commercial viability. For these reasons, concept 4 was considered the most suitable design for further investigation.

**Fig. 3:**
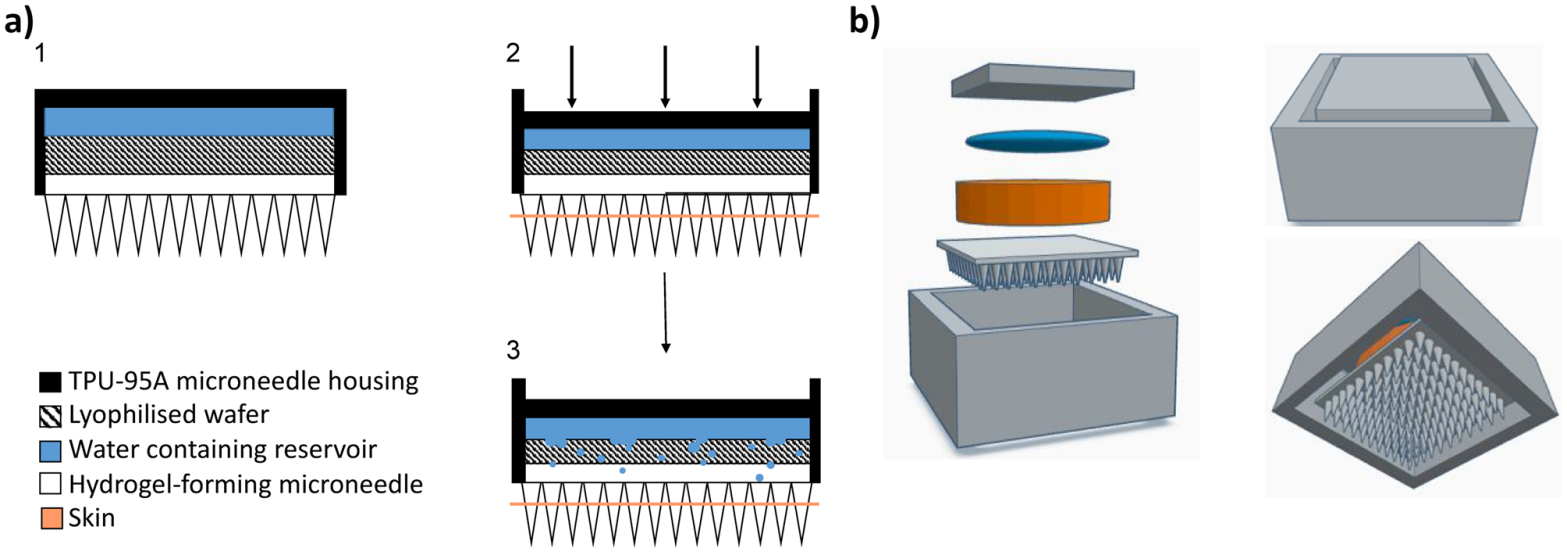
**a** Schematic representation of a one-step hydrogel-forming MAP insertion procedure. **b** MAP housing and lid design (concept 4)

Concept 4 displayed as a schematic in Fig. 3a demonstrates the application process of this design. Represented by the downward arrows, this concept is designed to compress the water containing reservoir, lyophilised wafer and hydrogel-forming MAP following the application of thumb pressure. It was envisaged that this application would produce two key actions, namely the insertion of MAPs into the skin beneath, in addition to a shorter wafer dissolution time due to the release of water from the reservoir situated above. This 2D concept was then designed using CAD software to produce a 3D construct. As shown in Fig. 3b, this concept consisted of two 3D printed components, the MAP housing and lid. With this design, the MAP would be set inside the housing and a lyophilised wafer placed on top. The water-filled reservoir would then be folded over once, positioned above the wafer and secured in place with a 3D printed lid.

### Fabrication of 3D printed MAP housing

**Fig. 4:**
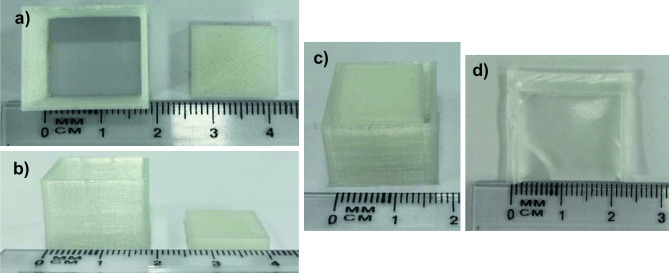
Images of 3D printed TPU 95a MAP applicator: **a** top view and **b** side view. **c** Complete MAP applicator containing a hydrogel-forming MAP, lyophilised drug containing wafer and Parafilm® M water reservoir. **d** Image of water (600 μL) retained within a Parafilm® M reservoir

The MAP housing, composed of TPU-95A, had internal dimensions 17 × 17 × 15 mm and a wall thickness of 2 mm, with a 15 × 15 × 3-mm lid (Fig. 4a, b). As shown in Fig. 4c, the dimensions of the MAP applicator were chosen to neatly accommodate the MAP, lyophilised wafer and water containing reservoir.

### Fabrication of Parafilm® M water-containing reservoirs

To achieve a watertight reservoir, each side of the Parafilm® M films was heat sealed for 10 s. A number of water loadings were then tested, with 600 μL proving to be the maximum volume that could be added (Fig. 4d). Using the same dimensions, 200 μL and 50 μL of water were also successfully loaded into the Parafilm® M reservoirs.

### Point of fracture and water release from Parafilm® M reservoirs

**Fig. 5:**
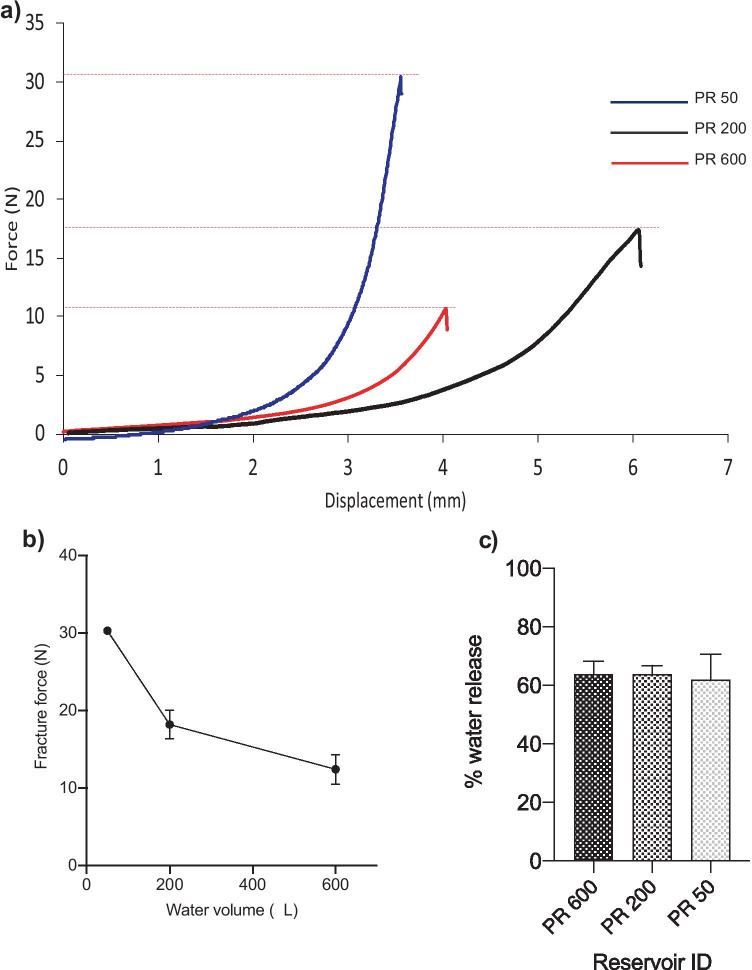
**a** Fracture point for three Parafilm® M reservoirs (PR 50, PR 200, PR 600) expressed as force vs displacement. **b** Fracture point vs water volume for PR 50, PR 200 and PR 600. **c** Percentage water released from PR 50, PR 200 and PR 600 after the fracture point had been reached. Mean ± SD. *n* = 3

It has previously been shown that patients exert a mean force of 30 N when applying a MAP to the skin after reading the associated patient information leaflet and following appropriate instruction from a pharmacist [24]. The point of Parafilm® M reservoir fracture was subsequently recorded to ensure that the force required to fracture the reservoir, and thus, release the required water volume, was comparable to the mean insertion force. The results presented in Fig. 5a, b indicate that increasing the volume of water encased within the Parafilm® M reservoir decreased the force required for reservoir fracture, although this is not a linear relationship. The force required for reservoir fracture was 12.41 ± 1.91 N, 18.18 ± 1.83 N and 30.27 ± 0.39 N for PR 600, PR 200 and PR 50, respectively. As expected, the reservoirs ruptured along the seams rather than in the body of the Parafilm® M. Importantly, all reservoirs produced a distint ‘pop’ sound upon rupture.

The difference in mass between a single 3D printed housing and lid before and after compression of PR 600, PR 200 and PR 50 was used to quantify the volume of water released from each individual reservoir (Fig. 5c). Interestingly, there was no significant difference in percentage water release between each reservoir (*p* > 0.05), with PR 600, PR 200 and PR 50 resulting in 63.86 ± 4.45%, 63.92 ± 2.85% and 62 ± 8.72% release, respectively.

### ‘Super-swelling’ hydrogel–forming MAP insertion into an artificial skin membrane using the unique feedback design

**Fig. 6:**
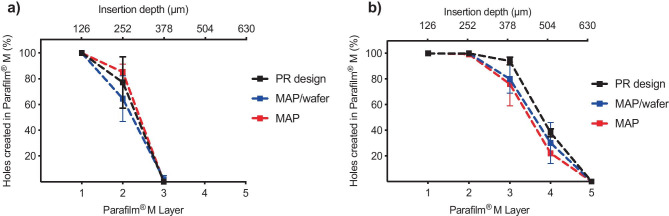
Percentage insertion of **a** 400 µm MAPs and **b** 600 µm MAPs using 3 different setups into 8 layers of Parafilm® M following application of a 30-N force for 30 s. This included MAP alone, MAP/wafer and PR design

Needle insertion into Parafilm® M was tested with three different MAP setups namely, MAP alone, MAP + wafer and MAP + wafer + PR 50 (PR design). To determine the robustness of a one-step application using a 30-N force, two different MAP types composed of different needle heights, namely 400 µm and 600 µm were tested. As shown in Fig. 6a, using a 400-µm MAP, 100% needle insertion into the first layer of Parafilm® M was observed with all three setups. The MAP alone displayed the greatest percentage insertion into the second layer; however, there was no significant difference between all three setups in this layer (*p* = 0.4516). Furthermore, no significant difference was observed in the third Parafilm® M layer (*p* = 0.7316). Assuming that successful insertion is equivalent to greater than 20% needle insertion in each layer, the needles within a 400-µm MAP reside between the 2nd and 3rd layers, equating to an insertion depth of 252–378 µm. As shown in Fig. 6b, 100% insertion in layers 1 and 2 was observed with all four setups using 600 µm MAPs. Although the PR design displayed the highest percentage insertion in layer 3, there was no significant difference between all three setups (*p* = 0.2527). Again, in layer 4, there was no significant difference in needle insertion between all three setups (*p* = 0.3912), with all three designs producing > 20% insertion within this layer.

#### ‘Super-swelling’ hydrogel–forming MAP in vitro swelling capacity using water-containing reservoirs

**Fig. 7:**
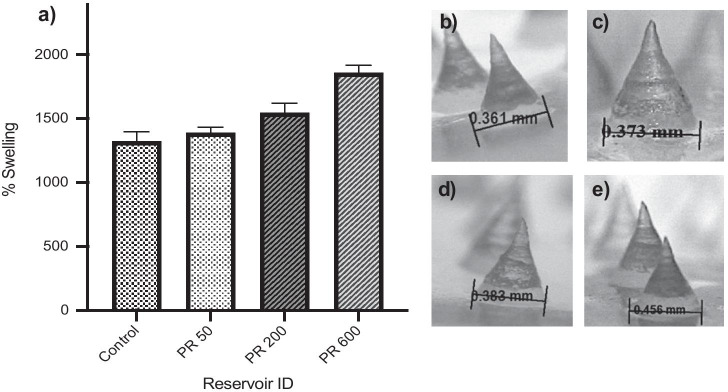
**a** Percentage swelling of each MAP following the addition of water from three Parafilm® M reservoirs after insertion into dermatomed neonatal porcine skin for 24 h. Means ± SD. *n* = 3. **b** Light microscope images highlighting the MAP base diameter after skin insertion for 24 h following the addition of **b** 10 µL (control), **c** PR 50, **d** PR 200 and **e** PR 600

Following insertion into dermatomed (350 µm) neonatal porcine skin in vitro using Franz cell apparatus, ‘super-swelling’ MAPs were removed and the weight recorded. From this, the % swelling was calculated as shown in Fig. 7a. With respect to the control, a significantly greater % swelling was observed with both PR 200 (*p* = 0.0206) and PR 600 (*p* = 0.0006) after 24 h. No significant difference in % swelling was observed with PR 50 (*p* = 0.2652). To further examine the extent of MAP swelling within the Franz cell apparatus, the MAP base diameter was measured using a Leica EZ4W stereo microscope (Fig. 7b). As expected, there was no statistical difference in base diameter between the control and PR 50 (*p* = 0.0993). In contrast, the needle diameter was found to be significantly greater with both PR 200 (*p* = 0.0019) and PR 600 (*p* < 0.0001) with respect to the control.

### Ex vivo swelling capacity of a ‘super-swelling’ hydrogel–forming MAP

**Fig. 8:**
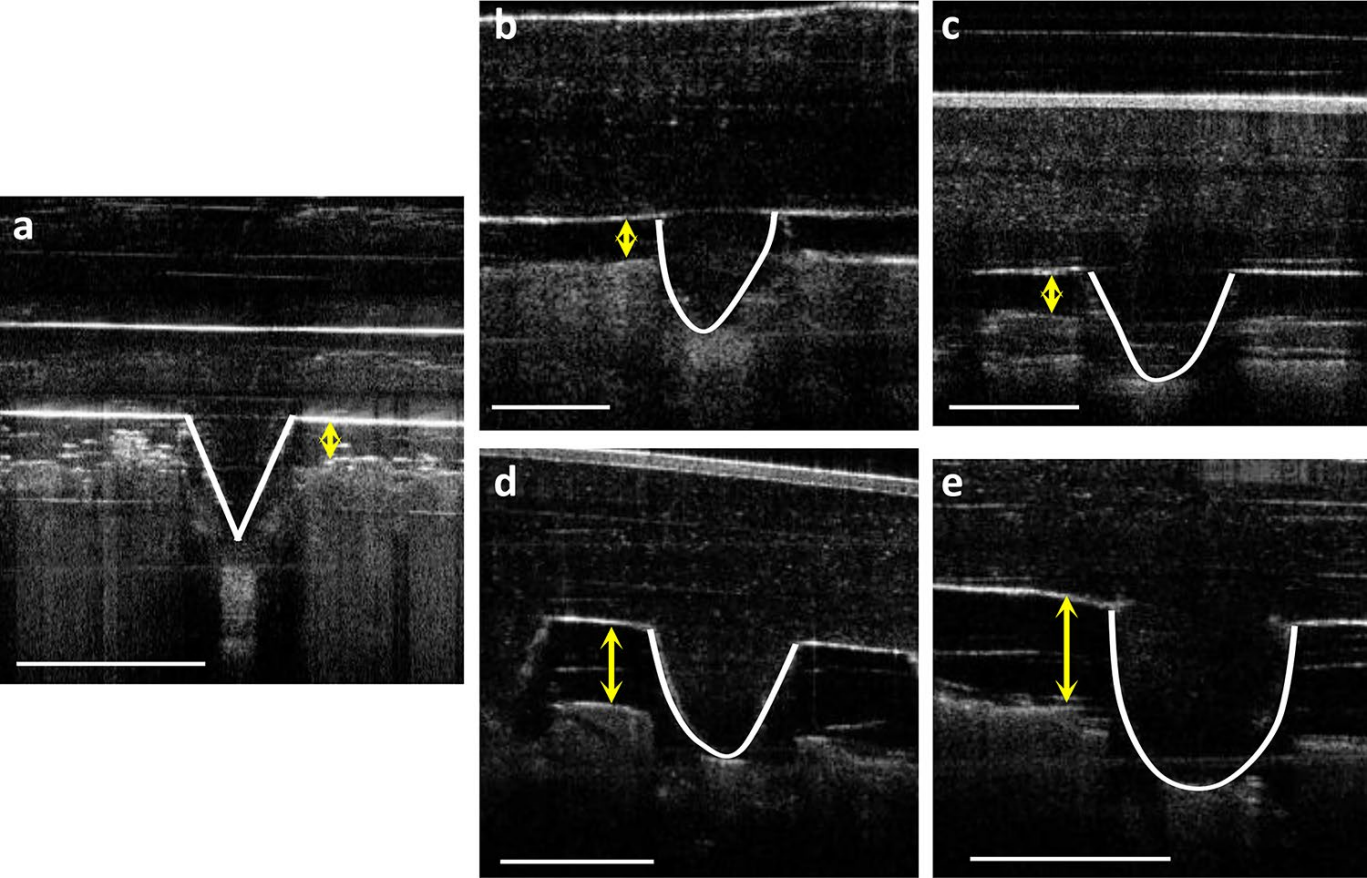
OCT images showing hydrogel-forming MAP insertion into excised full-thickness porcine skin using the 3D printed applicator device. **a** Needle insertion at *t* = 0 h. **b** Control after 24 h. **c** PR 50 after 24 h. **d** PR 200. **e** PR 600 after 24 h. Scale bar represents 0.5 mm

The swelling capacity of a ‘super-swelling’ MAP following insertion into excised neonatal porcine skin was examined using MAP base diameter and base plate/*stratum corneum* distance over 24 h. More specifically, this was performed using OCT imaging as shown in Fig. 8. Upon visual examination, it is clear that there was a positive correlation between the volume of water released and the MAP base diameter. As expected, an increase in the volume of water released resulted in an increase in MAP base diameter. This was also observed in vitro (Fig. 7). As detailed in Table 1, a significant difference in MAP base diameter with PR 200 and PR 600 was observed when compared to the control (*p* < 0.05). On the contrary, no significant difference in MAP base diameter between the control and PR 50 was observed *(p* = 0.1581). A similar trend was observed with the base plate/*stratum corneum* distance. Again, the difference between the control and PR 50 was deemed statistically insignificant (*p* = 0.3539). By comparing the base plate/*stratum corneum* distance for PR 200 and PR 600, it is clear that a larger MAP diameter has an adverse effect on MAP insertion.

**Table 1:** MAP base diameter and base plate/*stratum corneum* distance for 4 different water volumes following insertion into full-thickness neonatal porcine skin for 24 h. Means ± SD., *n* = 3

Reservoir ID	MAP base diameter (µm)	% difference relative to control	Base plate/S*tratum corneum* distance (µm)	% difference relative to control
Control	338 ± 8	-	209 ± 10	-
PR 50	348 ± 7	3.05 ± 2.11	220 ± 15	4.90 ± 2.14
PR 200	379 ± 10	10.91 ± 4.34	304 ± 6	31.24 ± 2.06
PR 600	423 ± 4	20.16 ± 2.31	437 ± 16	52.15 ± 0.62

### In vitro permeation of FITC-dextran 10 kDa and fluorescein sodium

**Fig. 9:**
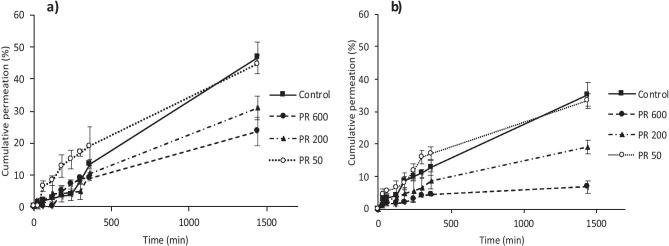
**a** In vitro permeation profile of fluorescein sodium through dermatomed (350 µm) neonatal porcine skin over 24 h following a one-step application process using different water-containing reservoirs. Means ± S.D., *n* = 3. **b** In vitro permeation profile of FITC-dextran 10 kDa subjected to the same study conditions. Means ± S.D., *n* = 3

The permeation profiles of fluorescein sodium and FITC-dextran 10 kDa following the release of three different volumes of water in vitro were compared against the control (Fig. 9a, b). In this case, the control represented the current method of application, in which 10 µL of water was added to the MAP baseplate, before a drug containing lyophilised wafer was placed on top. This simple, yet effective process, provides adhesion between the MAP and the drug layer. Notably, 10 µL of water was also incorporated into a PR, however, even upon application of a force much greater than 30 N, this system did not rupture. As shown in Fig. 9a, PR 600 resulted in 23.71 ± 4.46% fluorescein sodium permeation over 24 h, significantly lower than the control (47.53 ± 4.87%) (*p* = 0.0037). In addition, PR 200 also resulted in a significantly lower cumulative permeation (31.02 ± 3.73%) compared to the control (*p* = 0.0103). In contrast, no significant difference in permeation after 24 h was observed between PR 50 and the control (*p* = 0.2526). Additionally, the *f*_2_ factor was calculated to compare all permeation profiles (Eq. 2), using the 10 µL control as a reference (*R*_t_). PR 50 had an *f*_2_ value of 61, with *f*_2_ < 50 for both PR 200 and PR 600.

As shown in Fig. 9b, FITC-dextran 10 kDa produced an almost identical permeation profile to that of fluorescein sodium. Again, PR 600 performed poorly, with only 6.70 ± 1.84% permeation after 24 h and *f*_2_ < 50. PR 200 resulted in greater permeation (19.03 ± 1.96%) after 24 h; however, this was still deemed to be significantly lower than the control (*p* = 0.0003). Conversely, no significant difference in permeation was observed between PR 50 and the control, equivalent to 34.45 ± 2.29% and 35.28 ± 3.71%, respectively (*p* = 0.6400). This was confirmed with an *f*_2_ value of 84, further proving that the % cumulative permeation for both the control and PR 50 can be considered comparable.

## Discussion

Patient acceptance is perhaps one of the most important challenges to overcome within the pharmaceutical industry as it ultimately determines the success or failure of a novel drug delivery product. Therefore, with the end user at the forefront of this study, several alternative methods to enhance the patient experience were considered. The designs had two key aspects in mind, namely improving skin insertion consistency through a feedback mechanism and enhancing drug delivery by increasing the dissolution rate of the model drug–loaded lyophilised wafer.

It is widely recognised that for MAPs to become commercially viable, patients will require a feedback mechanism to confirm successful skin insertion [19]. One possible hydrogel-forming MAP insertion feedback mechanism that has previously been investigated utilised a pressure-indicating sensor film [19]. Here, a low-cost Pressurex-micro® Green colour changing film, producing a red impression following the application of pressure by the thumb was used. In all subjects, a colour change following MAP application was observed, showing that sufficient force was applied to the MAP for penetration into the skin. Notably, 75% of the subjects expressed a preference for this particular feedback mechanism. This highlights that confirmation of correct use is an important aspect in gaining both patient and prescriber acceptance. Considering an alternative approach in this particular study, a unique feedback mechanism involving a water-filled Parafilm® M reservoir was investigated. Using this design, it is anticipated that by hearing the distinctive ‘pop’ sound and by feeling the reservoir rupturing, this would provide a form of reassurance to a patient that the MAP has been successfully inserted into the skin. This multisensory approach could also have important benefits for patients with visual or hearing impairments. To this end, the ultimate aim of this study was to design a water-filled reservoir that was capable of rupturing upon application of 30 N, the average force exerted by a patient during MAP application after reading a patient information leaflet and receiving specific instruction from a pharmacist [24]. In doing so, patients can be instructed to apply a force until they feel/hear the fracture of the reservoir. Importantly, as Parafilm® M is a non-toxic substance which is malleable and can be heat-sealed easily, it was considered a suitable material for manufacture of the water-filled reservoir in this study.

In this proof-of-concept study, a range of water volumes were added to the Parafilm® M reservoirs to investigate the effects of water volume on reservoir fracture force, needle swelling and in vitro permeation of two model compounds. A maximum volume of 600 µL could be added to the reservoir, which was necessary to comfortably fit inside the 3D printed housing (Fig. 3c). The volume of water released from the reservoir at the point of rupture was consistently ~ 60% (Fig. 4b). All three reservoirs (containing 600 µL, 200 µL and 50 µL) successfully fractured upon application of a 30-N force. As PR ruptured at the seams rather than in the main body, it is clear that the dimensions in addition to the water volume have a direct influence on fracture force. Therefore, it can be assumed that a greater force would be required to fracture the reservoir if the seam width increased. Nevertheless, the PR dimensions chosen in this study have shown that a patient would be able to successfully fracture all three reservoirs using manual thumb pressure. Interestingly, PR 50 ruptured at exactly 30 N; therefore, this particular design certainly has the required properties to serve as a viable feedback mechanism.

Within this study, it was imperative that inclusion of a water reservoir would not affect needle insertion. This is because incomplete needle insertion prevents interstitial fluid uptake by the hydrogel-forming MAP and thus prevent drug within the lyophilised wafer from dissolving and diffusing across the imbibed MAP into the dermal microcirculation for systemic uptake. Notably, the control in this study has been successfully and reproducibly used by human volunteers to insert hydrogel-forming MAPs into their skin following appropriate instructions [16]. Importantly, there was no significant difference in MAP insertion depth between the control (no Parafilm® M reservoir) and the MAP situated within the 3D printed housing with the addition of the Parafilm® M reservoir (*p* > 0.05). This permitted in vitro testing of the novel feedback system as it was anticipated that the delivery of the model compounds fluorescein sodium and FITC dextran 10 kDa would be enhanced by the inclusion of the water reservoir system.

In addition to the convenience offered by this unique feedback design, it was also hypothesised that inclusion of the Parafilm® M reservoir system would increase the rate of model compound permeation into the skin. This is because fluid from the reservoir would be released immediately upon MAP insertion thus eliminating the delay between needle insertion and uptake of interstitial fluid. Theoretically, this would increase the rate of wafer dissolution, needle swelling and therefore, the rate of model compound diffusion across the imbibed needles into the skin. However, this was found not to be the case; there was a negative correlation observed between water release and model compound permeation after 24 h. It became clear that the addition of PR 200 and PR 600 was associated with a significantly greater % swelling of the MAP both in vitro and ex vivo*.* This adversely affected skin insertion, as indicated by the baseplate/*stratum corneum* distance (Table 1), with the increase in needle diameter causing the MAP to push out of the skin. By using a range of water volumes, it has been shown that PR 50 provided an optimal level of hydration for effective drug reservoir dissolution and skin insertion. Importantly, after 24 h, the % permeation between PR 50 and the control was comparable. This provides additional confirmation that PR 50 did not adversely affect skin permeation over the 24 h study time.

This study has also provided further evidence that hydrogel-forming MAPs can deliver high drug doses across the *stratum corneum* using a 1-cm^2^ patch. Here, up to 50% of fluorescein sodium, a low MW hydrophilic compound, was delivered in vitro. In addition, the cumulative permeation of FITC-dextran 10 kDa was ~ 38%. This proves that both compounds can be delivered in milligram levels, a distinct advantage over traditional transdermal patches. Furthermore, the cumulative permeation of both fluorescein sodium and FTIC-dextran 10 kDa has not reached a plateau after 24 h in vitro. Therefore, if this MAP was left in place for longer, it is assumed that even higher doses would be delivered. This is certainly achievable given that transdermal patches can be left in place for up to 7 days post application. Given that the control is the current method used for both in vitro and in vivo testing, the water-filled reservoir used in this study certainly indicates that the use of this hydrogel-forming MAP system has the potential to deliver high drug doses while ensuring consistent skin insertion through the feedback mechanism. Additionally, much larger MAPs may be required to deliver therapeutically relevant doses of low potency drugs [30]. In this instance, the unique feedback mechanism described in this study could be adapted to suit a larger patch size by simply increasing the dimensions and water volume of the reservoir.

## Conclusion

In this proof-of-concept study, a novel feedback mechanism for drug delivery using a hydrogel-forming MAP has been developed. Alongside the creation of a feedback mechanism, it was predicted that the use of a water-filled reservoir would in fact enhance drug permeation through the viable skin layers. While previous studies have shown that hydrogel-forming MAPs can successfully deliver a range of compounds, no such studies have examined the effect of increasing water volume on skin permeation. This study has shown that although increasing the water volume can indeed decrease the dissolution time of the drug containing reservoir, it ultimately has an adverse impact on MAP insertion. Using a 50-µL water-filled reservoir, which ruptured upon application of a 30-N force, up to 50% of fluorescein sodium and FITC-dextran 10 kDa, corresponding to milligram levels, was delivered in vitro. This design has demonstrated an ability to provide a suitable feedback mechanism; however, it can only be regarded as successful if it achieves patient acceptance. Therefore, the next step in this study will consider the views and opinions of human subjects.

## Data Availability

All data and materials support the claims in this manuscript and comply with field standards.

## Declarations

### Disclaimer

The companies had no role in the design of the manuscript, in the collection, analyses or interpretation of the various studies reviewed, in the writing of the manuscript or in the decision to publish.
